# The impact of pulmonary tuberculosis on SARS-CoV-2 infection: a nationwide cohort study

**DOI:** 10.3389/fmed.2024.1416197

**Published:** 2024-09-04

**Authors:** Sang Hwan Lee, Yun Jin Kim, Jaehoon Oh, Hyunggoo Kang, Kyung Hun Yoo, Byuk Sung Ko, Tae Ho Lim, Bo-Guen Kim, Hyun Lee, Sang-Heon Kim, Jang Won Sohn, Ho Joo Yoon, Hayoung Choi, Yongil Cho, Dong Won Park

**Affiliations:** ^1^Department of Emergency Medicine, Hanyang University College of Medicine, Seoul, Republic of Korea; ^2^Biostatistical Lab, Medical Research Collaborating Center, Hanyang University, Seoul, Republic of Korea; ^3^Division of Pulmonary Medicine and Allergy, Department of Internal Medicine, Hanyang University College of Medicine, Seoul, Republic of Korea; ^4^Division of Pulmonary, Allergy, and Critical Care Medicine, Department of Internal Medicine, Hallym University Kangnam Sacred Heart Hospital, Seoul, Republic of Korea

**Keywords:** COVID-19, pulmonary tuberculosis, tuberculosis, mortality, coinfection

## Abstract

**Background:**

The interaction between COVID-19 and tuberculosis (TB) is not yet fully understood, and large-scale research on the mortality outcome of such dual infection has been limited. This study aimed to investigate the impact of PTB on mortality among patients with COVID-19 within a Korean population by conducting an extensive analysis of a nationwide large dataset.

**Method:**

We investigated the mortality and disease severity among COVID-19 patients who had PTB in South Korea. This study analyzed 462,444 out of 566,494 COVID-19 patients identified between January 2020 and December 2021.

**Result:**

A total of 203 COVID-19 with PTB patients and 812 matched COVID-19 without PTB were analyzed using 1:4 propensity score matching. COVID-19 patients with PTB exhibited higher in-hospital mortality (odds ratio (OR) 3.02, 95% confidence interval (CI) 1.45–6.27, *p*-value = 0.003) and were at increased risk of requiring conventional oxygen therapy (OR 1.57, 95% CI 1.10–2.25, *p*-value = 0.013) as well as high flow nasal cannula (HFNC) or noninvasive ventilation (NIV) oxygen therapy (OR 1.91, 95 CI 1.10–3.32, *p*-value = 0.022) compared to those without PTB. Compared to matched COVID-19 without PTB, co-infected patients showed increased mortality rates across various timeframes, including during hospitalization, and at 30 day and 90 day intervals. In-hospital mortality rates were particularly elevated among women, individuals with malignancy, and those with lower incomes. Furthermore, the increased in-hospital mortality among PTB patients persisted irrespective of the timing of TB diagnosis or vaccination status against COVID-19.

**Conclusion:**

We suggest that physicians be aware of the risk of mortality and severity among COVID-19 patients with PTB; coinfection with COVID-19 is a critical situation that remains to be further explored and needs more attention in countries with an intermediate to high PTB burden.

## Introduction

The COVID-19 pandemic is a worldwide health issue. Previous studies have investigated factors, such as comorbidities, that influence the severity of COVID-19 in patients (1–7). Among these factors, several lung diseases have been identified as risk factors for disease severity and mortality. For example, patients with chronic obstructive pulmonary disease (COPD) have higher rates of intensive care unit (ICU) admission and mortality than non-COPD patients when infected with COVID-19 (5). Additionally, asthma patients with a history of acute exacerbations had higher mortality rates during COVID-19 infection (7).

In the context of the COVID-19 pandemic, pulmonary tuberculosis (PTB) has received renewed attention. PTB often presents with initial signs and symptoms similar to those of other viral infections, including COVID-19, although the prognosis and complications can vary. Cases of concurrent TB and COVID-19 remain relatively rare. However, the COVID-19 pandemic has significantly affected TB treatment. During this period, the provision of and access to essential TB services faced immense challenges, likely affecting the diagnosis and treatment of TB. As a result, the number of new TB cases and TB-related mortality increased for the first time in decades, a trend observed recently after the early years of the COVID-19 pandemic (8). Despite the development of vaccines and antiviral drugs, the risk of COVID-19 has not completely been resolved. Previous studies have indicated that coinfection with COVID-19 and TB leads to increased mortality rates, although the number of enrolled patients was relatively small. An early study during the COVID-19 pandemic in Wuhan, China, revealed a high mortality rate among patients coinfected with COVID-19 and TB (9). Furthermore, a meta-analysis that reviewed case reports and case series revealed a significantly greater risk of death in patients coinfected with COVID-19 and TB than in those without TB (10). This meta-analysis included 36 studies but involved only 89 patients with COVID-19 and TB coinfection. More recently, a study using the Korean population investigated the effects of chronic respiratory diseases, including TB infection of the lung, on the risk and mortality of COVID-19 patients (6). This study revealed that TB infection was not a statistically significant factor influencing hospital mortality in COVID-19 patients. Therefore, to determine whether PTB patients are at increased risk when they are infected with COVID-19, additional evidence is needed, particularly from large-scale studies.

Therefore, the aim of this study was to describe the clinical characteristics of patients co-infected with COVID-19 and PTB. And we aimed to investigate the impact of PTB on mortality for matched cohorts of COVID-19 with and without PTB by conducting an extensive analysis of a large nationwide dataset from Korea. In addition, we investigated the impact of co-infection with PTB and COVID-19 on patient severity.

## Methods

### Data sources and study setting

This study utilized the Korea Disease Control and Prevention Agency-COVID-19-National Health Insurance Service (K-COV-N) cohort, which is a database comprising national COVID-19 information sourced from the Korea Center for Disease Control and Prevention Agency (KCDC) and medical data from the National Health Insurance Service (NHIS). This database includes both hospitalized and non-hospitalized COVID-19 patients. The K-COV-N cohort includes a comprehensive health database with extensive demographic information (e.g., age, sex, income level reflected by insurance premium percentiles), date of death, and claims data (e.g., diagnoses and procedure codes). This includes claims for procedures such as nasal prongs, high-flow nasal cannula (HFNC), non-invasive ventilation (NIV), and mechanical ventilation. Additionally, the dataset provides the official date of SARS-CoV-2 infection, as well as COVID-19 vaccination status and type, all sourced from the Korea Disease Control and Prevention Agency (KDCA). The diagnoses were categorized using the International Classification of Diseases, 10th Revision (ICD-10) codes.

We conducted a retrospective cohort study using the K-COV-N cohort from January 2015 to December 2021. This study focused on adult patients aged 20 years and older because pulmonary tuberculosis cases are relatively rare in this age group, and they generally experience fewer lower mortality rates and complications related to COVID-19. Consequently, individuals younger than 20 years were excluded from the cohort database. Additionally, we determined that multiple COVID-19 infections (more than once) could impact complications and mortality. Therefore, patients who had two or more COVID-19 infections were excluded in this study. HIV patients were excluded because they had a high risk of death, which could lead to biased results in mortality. Additionally, to focus on the impact of PTB, which is presumed to be related to mortality and the severity of COVID-19 pneumonia, patients with extrapulmonary TB were excluded.

This study was approved by the institutional review board (IRB) of Hanyang University Hospital (IRB No. HYUH 2023-02-033). The requirement for informed consent from the participants was waived because the NHIS database was constructed after anonymization.

### Definitions

In South Korea, the NHIS operates a rare intractable disease (RID) registration program. To qualify for enrollment in the RID program, patients must meet the diagnostic criteria provided by the NHIS for each RID, and assessments must be approved by specialized physicians. The diagnostic codes are defined according to the ICD-10, and a specific NHIS code (V code) is assigned in the RID database. In this study, PTB infection was defined as one or more visits with the relevant ICD-10 code for PTB (A15 or A16), along with specific NHIS codes (V206, V246, and V000).

Our objective was to assess the impact of PTB coinfection on COVID-19 outcomes; thus, we exclusively considered cases of coinfection where PTB was present before or concurrently (within the same week) with COVID-19 infection. Patients with PTB were identified as those who had visited the hospital for at least one PTB treatment session within the 60 days preceding their COVID-19 infection.

### Main outcomes and measures

The primary outcome of this study was in-hospital mortality. In-hospital mortality was defined as hospitalization within 7 days of a COVID-19 diagnosis and subsequent death during the hospital stay. Additionally, 30 day mortality, 90 day mortality, and disease severity were investigated as secondary outcomes. The classification of disease severity was determined based on respiratory support, with reference to the therapeutic management guidelines for adult COVID-19 patients provided by the National Institutes of Health (11). Disease severity was subsequently categorized into three levels: (1) conventional oxygen therapy, (2) high-flow nasal cannula (HFNC) or non-invasive ventilation (NIV), and (3) mechanical ventilation. Conventional oxygen therapy refers to oxygen supplementation that does not include HFNC, NIV, or mechanical ventilation, such as the use of nasal prongs or simple oxygen masks.

### Variables

The baseline patient demographic data obtained from the NHIS claims database were age group, sex, type of insurance, and diagnosis of comorbidities according to ICD-10 codes. We calculated the Charlson Comorbidity Index (CCI), which encompasses 22 comorbid conditions, including cardiovascular disease and malignancy (12, 13). Because HIV patients were excluded from this study, the scores for HIV were omitted from the CCI score. Income levels were categorized into four quartiles. We investigated the history of COVID-19 vaccination prior to COVID-19 infection.

### Statistical analysis

Categorical variables are presented as numbers and percentages. The normality of the continuous variables was tested using the Anderson-Darling test. Continuous variables with a normal distribution are presented as medians and 25th to 75th percentiles. Student’s *t* test or the Wilcoxon rank sum test was performed depending on the results of the normality test. Fisher’s exact test was performed when comparing the categorical variables between two groups.

To address potential confounders in the primary prespecified analysis, we employed propensity score matching to ensure a fair comparison between patients with PTB and matched non-PTB controls. For each PTB patient, we identified matched non-PTB controls through a 1:4 matching process, where each PTB patient was matched with up to four non-PTB controls based on their propensity scores. These propensity scores estimate the probability of having PTB given a set of observed covariates. The variables used to calculate these propensity scores included age, sex, income quartile, COVID-19 vaccination status, and each disease listed in the CCI. The exact age in 1 year increments in years was used for matching. We performed the matching using a greedy algorithm with a caliper of 0.25 standard deviations of the logit of the propensity score. To evaluate the effectiveness of the matching process, we assessed the balance between the matched groups in terms of baseline characteristics using standardized differences. Standardized differences after matching are presented in Table 1, while those before matching are shown in Supplementary Table S1. A reduction in these standardized differences indicates that the matching process effectively controlled for confounding variables and improved the comparability between the PTB and non-PTB groups.

**Table 1:** Baseline characteristics after propensity-score matching.

	COVID-19 patients		Standardized difference
	PTB cases (*N* = 203)	Non-PTB case (*N* = 812)	
Age (years), median (25–75%)	68 (55–80)	68 (56–80)	0.013
Age group, *n* (%)			<0.001
20–29	14 (6.9)	56 (6.9)	<0.001
30–39	11 (5.42)	44 (5.42)	<0.001
40–49	8 (3.94)	32 (3.94)	<0.001
50–59	30 (14.78)	120 (14.78)	<0.001
60–69	44 (21.67)	176 (21.67)	<0.001
70–79	43 (21.18)	172 (21.18)	<0.001
≥80	53 (26.11)	212 (26.11)	<0.001
Sex, *n* (%)			<0.001
Male	127 (62.56)	479 (58.99)	0.073
Female	76 (37.44)	333 (41.01)	0.073
Income			<0.001
Quintile 1 (lowest)	57 (28.08)	247 (30.42)	0.052
Quintile 2	41 (20.2)	126 (15.52)	0.122
Quintile 3	51 (25.12)	217 (26.72)	0.037
Quintile 4	54 (26.6)	222 (27.34)	0.017
Previous vaccination, *n* (%)	90 (44.33)	310 (38.18)	0.125
Charlson comorbidity index, median (25–75%)	8 (3–11)	6 (3–11)	0.113
Hypertension	108 (53.2)	377 (46.43)	0.136
DM without chronic complication	128 (63.05)	495 (60.96)	0.043
DM with chronic complication	36 (17.73)	117 (14.41)	0.091
Peripheral vascular disease	58 (28.57)	202 (24.88)	0.084
Renal disease	19 (9.36)	67 (8.25)	0.039
Chronic pulmonary disease	147 (72.41)	557 (68.6)	0.084
Rheumatic disease	28 (13.79)	105 (12.93)	0.025
Dementia	40 (19.7)	128 (15.76)	0.103
Ulcers of the digestive system	96 (47.29)	351 (43.23)	0.082
Hemiplegia or paraplegia	14 (6.9)	51 (6.28)	0.025
Mild liver disease	143 (70.44)	565 (69.58)	0.019
Moderate or severe liver disease	5 (2.46)	19 (2.34)	0.008
Cerebrovascular disease	55 (27.09)	214 (26.35)	0.017
Congestive heart failure	77 (37.93)	281 (34.61)	0.069
Myocardial infarction	19 (9.36)	62 (7.64)	0.062
Malignancy	79 (38.92)	317 (39.04)	0.003
Metastatic solid tumor	13 (6.4)	49 (6.03)	0.015
Depression	51 (25.12)	184 (22.66)	0.058

PTB, pulmonary tuberculosis. DM, diabetes mellitus.

After propensity score matching, univariable logistic regression analyses for predicting mortality and disease severity were conducted. We confirmed the independence of errors was satisfied using the Durbin-Watson test and assessed no significant multi-collinearity using the Variance Inflation Factor. The results are presented as odds ratios (ORs) and 95% confidence intervals (CIs). The Kaplan–Meier method was used to generate survival curves, and the log-rank test was used to compare survival between the two groups. We have conducted analyses using log–log plots to evaluate the proportional hazards assumption visually. Using these plots, we confirmed that the survival curves were parallel, indicating that the proportional hazards assumption is valid.

In the subgroup analysis, the risk of in-hospital mortality was identified by comparing PTB patients with each matched non-PTB control patient group. To assess whether the vaccine has a protective effect in PTB patients with COVID-19, we conducted a multivariable logistic regression analysis. We adjusted for age, sex, income quartile, and CCI score to calculate the adjusted ORs (aORs) and 95% confidence intervals (CIs) for mortality and oxygen therapy. All analyses were conducted using SAS version 9.4 (SAS Institute, Cary, NC, United States). All tests were two-sided, and *p* values less than 0.05 were used to indicate statistical significance.

## Results

**Figure 1:**
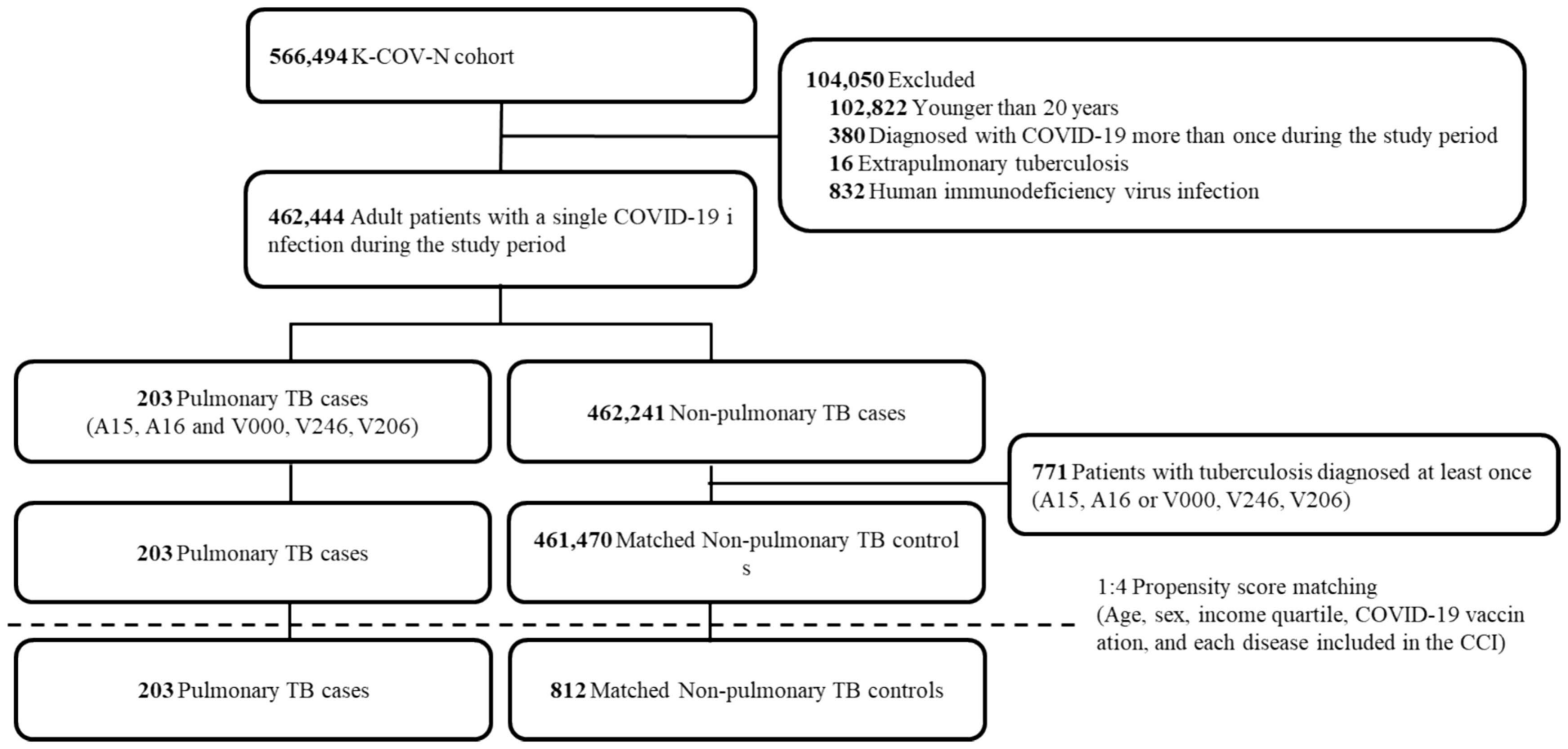
Flow chart of the study population. K-COV-N, Korea disease control and prevention agency-COVID-19-national health insurance service; TB, tuberculosis; CCI, Charlson comorbidity index.

We included 566,494 patients with a diagnosis of COVID-19 between January 2020 and December 2021 (Figure 1). After excluding 104,050 patients, 462,444 patients remained. We found 203 PTB-coinfected patients in the Korean general population with COVID-19 infection, and the prevalence of PTB-coinfected patients was 43.9 per 100,000 individuals infected with COVID-19. Before propensity score matching, there were significant differences in demographic and clinical characteristics between the COVID-19 patients with PTB and the COVID-19 patients without PTB (Supplementary Table S1). A total of 203 patients coinfected with COVID-19 and PTB were older than were those not coinfected (median 68 vs. 49 years), with a greater percentage being older than 70 years (47.3% vs. 11.9%) and having lower income levels. Additionally, a greater proportion of coinfected patients with COVID-19 and PTB had malignancies as comorbid conditions than did those without coinfection (38.9% vs. 9.5%). Nonetheless, the proportion of vaccinated individuals did not significantly differ between the two groups.

Through 1:4 propensity score matching, this study analyzed 203 patients with PTB and 812 matched non-PTB controls (Figure 1). Table 1 presents the baseline characteristics of the matched study population. The standardized differences were within the acceptable limits. In the matched population, approximately two-thirds were in their 60s, and about 60% were male. Around 40% of the patients had received the COVID-19 vaccine. The prevalence of chronic pulmonary disease, congestive heart failure, and malignancy among the patients was approximately 70, 35, and 40%, respectively.

Table 2 presents the mortality rate and disease severity of the study population. Among PTB patients, the mortality rate was greater than that among non-PTB controls at all time intervals (in-hospital, 6.40% vs. 2.22%; 0–30 days, 11.33% vs. 6.40%; 0–90 days, 15.27% vs. 7.88%). Among PTB patients, the proportion not receiving oxygen therapy was lower (60.59% vs. 73.15%, *p* < 0.001), and the proportion receiving oxygen therapy was greater (39.41% vs. 26.85%, *p* < 0.001). The prevalence of conventional oxygen therapy combined with HFNC therapy or NIV oxygen support was significantly greater than that of matched non-PTB controls (conventional oxygen therapy, 26.6% vs. 18.72%, *p* = 0.015; HFNC therapy or NIV oxygen support, 9.85% vs. 5.42%, *p* = 0.024). However, there were no significant differences in mechanical ventilation between the two groups (2.96% vs. 2.71%, *p* = 0.812).

**Table 2:** The proportion of mortality and disease severity in the study population after propensity score matching.

	COVID-19 patients		*p* value
	PTB cases (*N* = 203)	Matched non-PTB controls (*N* = 812)	
Mortality, *n* (%)			
COVID-19 in hospital	13 (6.40)	18 (2.22)	0.005
30 days	23 (11.33)	52 (6.40)	0.024
90 days	31 (15.27)	64 (7.88)	0.003
Severity, *n* (%)			
No oxygen	123 (60.59)	594 (73.15)	<0.001
Oxygen supply	80 (39.41)	218 (26.85)	<0.001
Conventional oxygen therapy^*^	54 (26.6)	152 (18.72)	0.015
HFNC oxygen or NIV	20 (9.85)	44 (5.42)	0.024
Mechanical ventilation	6 (2.96)	22 (2.71)	0.812

*Conventional oxygen therapy refers to oxygen supplementation that does not include HFNC, NIV, or mechanical ventilation, such as the use of nasal prongs or simple oxygen masks. PTB, pulmonary tuberculosis. HFNC, high flow nasal cannula; NIV, noninvasive ventilation.

**Figure 2:**
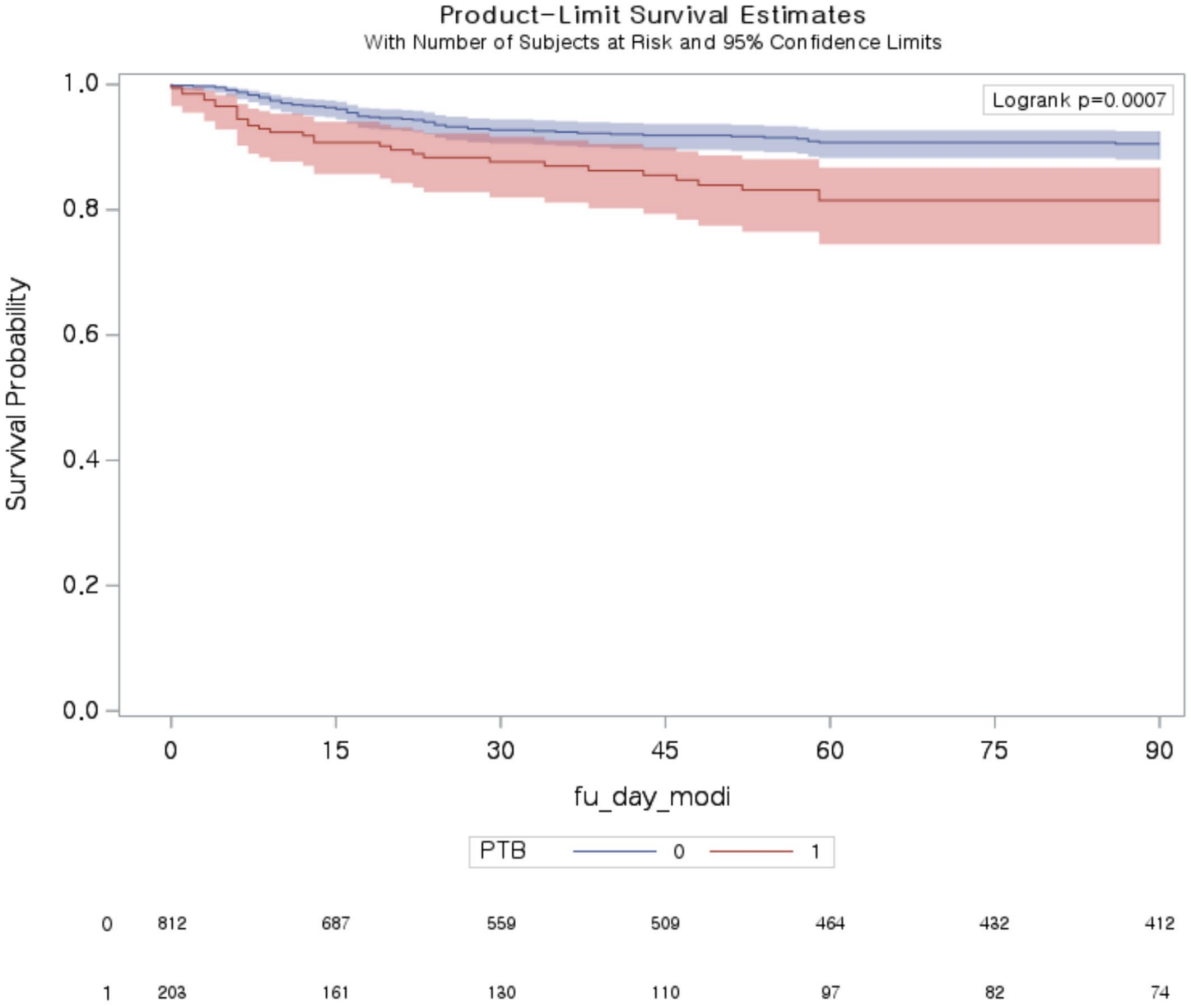
Ninety-day mortality among patients with COVID-19 with and without pulmonary tuberculosis. PTB, pulmonary tuberculosis.

Table 3 presents the ORs and 95% CIs of COVID-19 mortality and disease severity. Among patients with PTB, in-hospital mortality was significantly greater than that in non-PTB controls (OR 3.02 [95% CI, 1.45–6.27]). The median (Q1–Q3) hospital length of stay for PTB patients and control patients were 6 (4–13) and 8.5 (6–15) days, respectively. The mortality rate in the subsequent period was also greater in the PTB group than in the non-PTB control group (30 day mortality, OR 1.87 [95% CI, 1.11–3.13]; 90 day mortality, OR 2.11 [95% CI, 1.33–3.34]; Figure 2). Among patients with PTB, the odds ratio (OR) for no oxygen therapy was significantly lower than that for non-PTB controls (OR 0.56 [95% CI, 0.41–0.78]) (Table 3). Similarly, the ORs for conventional oxygen therapy and HFNC therapy or NIV oxygen support were significantly greater than those for non-PTB controls (conventional oxygen therapy, OR 1.57 [95% CI, 1.10–2.25]; HFNC therapy or NIV oxygen support, OR 1.91 [95% CI, 1.10–3.32]). However, there was no significant difference in mechanical ventilation between the two groups (OR 1.09 [95% CI, 0.44–2.73]).

**Table 3:** Logistic regression analysis of the impact of pulmonary tuberculosis on outcomes in COVID-19 patients after propensity score matching.

	OR (95% CI)	*p* value
Mortality		
COVID-19 in hospital	3.02 (1.45–6.27)	0.003
30 days	1.87 (1.11–3.13)	0.018
90 days	2.11 (1.33–3.34)	0.002
Severity		
No oxygen	0.56 (0.41–0.78)	<0.001
Oxygen supply	1.77 (1.29–2.44)	<0.001
Conventional oxygen therapy^*^	1.57 (1.10–2.25)	0.013
HFNC oxygen or NIV	1.91 (1.10–3.32)	0.022
Mechanical ventilation	1.09 (0.44–2.73)	0.848

*Conventional oxygen therapy refers to oxygen supplementation that does not include HFNC, NIV, or mechanical ventilation, such as the use of nasal prongs or simple oxygen masks. OR, odds ratio; CI, confidence intervals; HFNC, high flow nasal cannula; NIV, noninvasive ventilation.

The results of the subgroup analysis of in-hospital mortality are shown in Table 4. This study divided the time interval into 2 months to determine the impact of the time interval between PTB diagnosis and COVID-19 infection on the outcomes of patients coinfected with COVID-19 and PTB. The analysis revealed an increased risk of in-hospital mortality for PTB patients compared to for those without PTB, both for patients diagnosed with COVID-19 within 60 days of their PTB diagnosis (OR 3.55 [95% CI, 1.05–12.08]) and for those diagnosed more than 60 days after PTB diagnosis (OR 2.76 [95% CI, 1.11–6.89]). The risk significantly increased in patients aged ≥70 years (OR 3.08 [95% CI, 1.42–6.70]), female sex (OR 3.65 [95% CI, 1.32–10.14]), malignancy (OR 4.99 [95% CI, 1.75–14.21]), and lowest income (OR 7.15 [95% CI, 1.95–26.25]). Moreover, we found an increased risk of in-hospital mortality for PTB patients compared to non-PTB controls, regardless of their COVID-19 vaccination status (vaccinated individuals, OR 3.59 [95% CI, 1.02–12.68]; unvaccinated individuals, OR 2.87 [95% CI, 1.16–7.09]). The results of the multivariable logistic regression analysis conducted to assess whether the vaccine has a protective effect among 203 PTB patients with COVID-19 can be found in Supplementary Table S4. After adjusting for covariates, vaccinated patients were associated with 68% lower in-hospital mortality rate (aOR 0.32; 95% CI 0.01–1.31). However, due to the small sample size, the analysis lacked sufficient power to achieve statistical significance. Nonetheless, the vaccine was significantly associated with a threefold increased likelihood of not requiring oxygen therapy (aOR 3.01; 95% CI 1.51–5.97). Conversely, it was significantly associated with a one-fourth lower likelihood of requiring HFNC or NIV (aOR 0.24; 95% CI 0.08–0.75).

**Table 4:** Stratified analysis of the impact of pulmonary tuberculosis on COVID-19 patients compared to a matched non-tuberculosis control group.

	Odds ratio (95% confidence interval)
Interval between PTB and COVID-19 diagnosis^*^	
intervals ≥60 days (*N* = 145)	2.76 (1.11–6.89)
<60 days (*N* = 58)	3.55 (1.05–12.08)
*P for interaction*	0.747
Age	
≥70 years	3.08 (1.42–6.70)
<70 years	4.03 (0.25–64.93)
*P for interaction*	0.856
Sex	
Male	2.59 (0.90–7.42)
Female	3.65 (1.32–10.14)
*P for interaction*	0.6456
Malignancy	
Yes	4.99 (1.75–14.21)
No	1.85 (0.63–5.42)
*P for interaction*	0.195
Incomes	
Quintile 1 (lowest)	7.15 (1.95–26.25)
Quintile 2	1.56 (0.28–8.87)
Quintile 3	0.85 (0.10–7.42)
Quintile 4	3.47 (0.90–13.40)
*P for interaction*	0.365
COVID-19 vaccination	
Yes	3.59 (1.02–12.68)
No	2.87 (1.16–7.09)
*P for interaction*	0.777

PTB, pulmonary tuberculosis.

In addition, we performed the analyses for unmatched patients (Supplementary Tables S1–S3). Significant differences were observed between the two groups in terms of baseline characteristics, including age, sex, and CCI score. Consistent with the results after propensity score matching, there were also differences in mortality and disease severity. The mortality rate for PTB patients was greater than that for non-PTB controls at all time intervals.

The period of COVID-19 infection is important because infection variants and treatment guidelines can change over time, potentially affecting study outcomes. Therefore, we included the period of COVID-19 infection as a matching variable and conducted a sensitivity analysis to determine if the results remained consistent. We added the period of COVID-19 infection in months as a propensity score matching variable, along with exact age in 1 year increments, sex, income quartile, COVID-19 vaccination status, and CCI score. Since 1:4 matching could not be achieved for each disease listed in the CCI, we substituted the CCI score instead. Supplementary Tables S5, S6 correspond to the original analysis results presented in Tables 2, 3, respectively. The results were similar to those of the original analysis; however, conventional oxygen therapy did not show statistical significance in the sensitivity analysis. Nonetheless, when including the period of COVID-19 infection, the ORs for in-hospital mortality (OR 4.21 [95% CI, 1.92–9.22]) and 30 day mortality (OR 2.40 [95% CI, 1.41–4.11]) associated with PTB were higher compared to the original results.

## Discussion

In South Korea, a country with an intermediate burden of TB, the incidence of PTB among those infected with COVID-19 was 43.9 per 100,000, similar to that in the general population (14). Patients with PTB and COVID-19 coinfections were generally older, had lower incomes, and exhibited a greater incidence of comorbidities, including malignancies. Through a comprehensive analysis of a large nationwide dataset, we examined mortality and disease severity among COVID-19 patients with PTB in the Korean population. Compared to matched COVID-19 patients without PTB, coinfected patients had increased mortality rates across various timeframes. In particular, in-hospital mortality was more than three times higher in COVID-19 patients with PTB compared to those without PTB. 30 day and 90 day mortality rate were approximately twice as high in COVID-19 patients with PTB compared to those without PTB. The severity of COVID-19 was more pronounced in PTB patients. Specifically, the need for conventional oxygen therapy and HFNC or NIV oxygen support increased by 1.57 and 1.91 times, respectively. COVID-19 patients without PTB were about half as likely to require oxygen compared to those with PTB. Notably, in-hospital mortality rates were particularly elevated among women, individuals with malignancies, and individuals with lower incomes. Furthermore, the increase in in-hospital mortality among PTB patients persisted irrespective of the timing of TB diagnosis or vaccination status for COVID-19.

Several studies have explored the association between COVID-19 and TB infection. One study explained the shared immune response and the potential risk of coinfection between two diseases through transcriptomic evaluation (15). Both COVID-19 and TB infection exhibit an increase in the production of interferons and upregulation of response signatures, which are associated with severe disease and may contribute to disease progression and fatal outcomes (16, 17). Additionally, TB may exacerbate COVID-19 through impaired immune responses and increased expression of angiotensin-converting enzyme 2 receptors in respiratory epithelial cells, whereas COVID-19 pneumonia may accelerate the progression of TB. The present study described the features of PTB and COVID-19 coinfected patients and showed considerable value, as real-world data from a large population-based cohort study established the relationship between PTB and COVID-19 infection.

In the present study, we focused on patients with PTB before or concurrently (within the same week) with COVID-19. Coinfection with PTB and COVID-19 was categorized based on the timing of diagnoses for the two infections (15). These included cases where COVID-19 was diagnosed before PTB infection, cases where both infections were diagnosed simultaneously (including cases identified within the same week), and cases where PTB was diagnosed before COVID-19 infection. Previous research has shown that TB is frequently diagnosed concomitantly with or before COVID-19, and such dual infections could lead to a higher mortality rate (16, 17). Nonetheless, earlier investigations have included all such cases together without distinction. Our study focused on the role of PTB as a risk factor for the outcome of COVID-19 patients.

Despite the development of vaccines and antiviral treatments, COVID-19 has significantly disrupted crucial TB services, resulting in severe global health consequences. The gradual downward trend in new TB cases worldwide has been reversed, leading to an increase in TB mortality for the first time in recent decades (8, 18). The interaction between COVID-19 and TB has exacerbated the global TB epidemic and weakened National TB programs, similar to the historical impact of TB and HIV coinfection. This critical situation requires further exploration and additional attention in countries with intermediate to high TB burdens (19, 20). Notably, the COVID-PTB co-infected patient population in this study predominantly consisted of older individuals with numerous comorbidities, which is different from the findings of other countries, which typically involve younger patients in their 40s and 50s (16, 17, 21). This might be due to the prevalence of MTB in the studied countries and the heterogeneity of the inclusion criteria, such as HIV patients. These are the important features of the present study that deserve to be highlighted because they differentiate it from previous studies.

Furthermore, the present study elucidates the resource challenges associated with managing COVID-PTB-coinfected patients. Our data indicate that a significant portion of coinfected patients need oxygen therapy, including the use of HFNC or NIV, which leads to the demand for skilled healthcare professionals and adequate facilities to treat those with COVID-19-PTB coinfection suffering from respiratory failure. This requirement supports the adverse effects of the COVID-19 pandemic on TB services.

Our study has several limitations. First, we analyzed a total of 462,444 COVID-19 patients; however, the significance of certain subgroups (such as those receiving mechanical ventilation) was unclear due to the insufficient sample size. Second, our analysis was conducted before the emergence of the Omicron variant. Third, the use of claims data from the NHIS limited our ability to assess clinical details for patients with both PTB and COVID-19. Essential variables that might influence outcomes, such as the extent of PTB according to chest radiography and laboratory biomarkers, were not included in our study. Fourth, there may have been potential influences from unmeasured confounding factors that could have affected the results. Specifically, factors such as the severity of PTB, adherence to COVID-19 treatment protocols, and various social determinants of health were not assessed in our study. To address this concern, we included the period of COVID-19 infection as a confounding factor in the propensity score matching during our sensitivity analysis. This adjustment accounted for temporal variations in disease severity and treatment protocols. The results remained consistent with our original findings, indicating that the period of infection did not significantly alter the observed associations. Fifth, evidence indicates that COVID-19 reinfection increases the risk of death, hospitalization, and long-term health issues (22). While this study focused on the first episode of COVID-19 infection, further research is necessary to understand the impact of PTB in patients with reinfections. Sixth, the study population is specific to South Korea, which may limit the generalizability of our findings to other regions with different demographics, healthcare systems, or prevalence of PTB and COVID-19.

In conclusion, PTB was significantly associated with higher mortality rates and increased severity of certain oxygen support interventions among COVID-19 patients. PTB infection should be recognized as a risk factor for severe and fatal COVID-19 infection. Thus, physicians and public health authorities should be aware of the risk of concurrent PTB and COVID-19 infections.

## Data Availability

The datasets presented in this article are not readily available because National health insurance service data is stored on specific computers by the South Korean government and cannot be taken outside. Requests to access the datasets should be directed to joeguy@hanyang.ac.kr.
