# Supplementary material for: The impact of pulmonary tuberculosis on SARS-CoV-2 infection: a nationwide cohort study
Source: Front Med (Lausanne). 2024 Sep 4;11:1416197. doi: 10.3389/fmed.2024.1416197 (PMC11408221; doi:10.3389/fmed.2024.1416197)
Supplement: Supplementary file 1 [file Table_1.docx]

| **Supplementary table 1.** Baseline characteristics of study subjects before propensity score matching. | | | | |
| --- | --- | --- | --- | --- |
|  | COVID-19 patients | |  |  |
|  | PTB cases  (N =203) | Non-PTB case  (N =461,470) | P value | Standardized difference |
| Age (years), median (25%-75%) | 68 (55-80) | 49 (34-62) | <0.001 | 0.910 |
| Age group, n (%) |  |  | <0.001 |  |
| 20-29 | 14 (6.9) | 77,777 (16.85) |  | 0.311 |
| 30-39 | 11 (5.42) | 78,198 (16.95) |  | 0.372 |
| 40-49 | 8 (3.94) | 82,445 (17.87) |  | 0.459 |
| 50-59 | 30 (14.78) | 83,734 (18.15) |  | 0.091 |
| 60-69 | 44 (21.67) | 84,505 (18.31) |  | 0.084 |
| 70-79 | 43 (21.18) | 35,645 (7.72) |  | 0.390 |
| ≥80 | 53 (26.11) | 19,166 (4.15) |  | 0.644 |
| Sex, n (%) |  |  | <0.001 |  |
| Male | 127 (62.56) | 233,633 (50.63) |  | 0.243 |
| Female | 76 (37.44) | 227,837 (49.37) |  | 0.243 |
| Income |  |  | 0.315 |  |
| Quintile 1 (lowest) | 57 (28.08) | 106,047 (22.98) |  | 0.117 |
| Quintile 2 | 41 (20.2) | 106,599 (23.1) |  | 0.071 |
| Quintile 3 | 51 (25.12) | 113,786 (24.66) |  | 0.011 |
| Quintile 4 | 54 (26.6) | 135,038 (29.26) |  | 0.059 |
| Previous vaccination, n (%) | 90 (44.33) | 216,649 (46.95) | 0.456 | 0.053 |
| Charlson comorbidity index, median (25%-75%) | 8 (3-11) | 2 (1-5) | <0.001 | 0.970 |
| Hypertension | 108 (53.2) | 133,707 (28.97) | <0.001 | 0.508 |
| DM without chronic complication | 128 (63.05) | 134,107 (29.06) | <0.001 | 0.725 |
| DM with chronic complication | 36(17.73) | 31,181 (6.76) | <0.001 | 0.339 |
| Peripheral vascular disease | 58(28.57) | 69,092 (14.97) | <0.001 | 0.334 |
| Renal disease | 19(9.36) | 12,775 (2.77) | <0.001 | 0.279 |
| Chronic pulmonary disease | 147(72.41) | 196,354 (42.55) | <0.001 | 0.634 |
| Rheumatic disease | 28(13.79) | 36,211 (7.85) | 0.002 | 0.192 |
| Dementia | 40(19.7) | 27,552 (5.97) | <0.001 | 0.419 |
| Ulcers of the digestive system | 96(47.29) | 144,872 (31.39) | <0.001 | 0.330 |
| Hemiplegia or paraplegia | 14(6.9) | 5,859 (1.27) | <0.001 | 0.287 |
| Mild liver disease | 143(70.44) | 176,119 (38.16) | <0.001 | 0.685 |
| Moderate or severe liver disease | 5(2.46) | 1,977 (0.43) | 0.005 | 0.171 |
| Cerebrovascular disease | 55(27.09) | 47,437 (10.28) | <0.001 | 0.442 |
| Congestive heart failure | 77(37.93) | 47,908 (10.38) | <0.001 | 0.680 |
| Myocardial infraction | 19(9.36) | 11,584 (2.51) | <0.001 | 0.293 |
| Malignancy | 79(38.92) | 44,028 (9.54) | <0.001 | 0.730 |
| Metastatic solid tumor | 13(6.4) | 4,606 (1.0) | <0.001 | 0.289 |
| Depression | 51(25.12) | 56,928 (12.34) | <0.001 | 0.332 |

Abbreviations: PTB, pulmonary tuberculosis; DM, diabetes mellitus.

| **Supplementary table 2.** The proportion of mortality and disease severity in the study population before propensity score matching.. | | | |
| --- | --- | --- | --- |
|  | COVID-19 patients | | p value |
|  | PTB cases  (N =203) | Non-PTB controls  (N =461,470) |  |
| **Mortality,** n (%) |  |  |  |
| COVID-19 in hospital | 13 (6.40) | 2,223 (0.48) | <0.001 |
| 30 days | 23 (11.33) | 5,216 (1.13) | <0.001 |
| 90 days | 31 (15.27) | 7,462 (1.62) | <0.001 |
| **Severity,** n (%) |  |  |  |
| No oxygen | 123 (60.59) | 415,210 (89.98) | <0.001 |
| Oxygen supply | 80 (39.41) | 46,260 (10.02) | <0.001 |
| Conventional oxygen therapy^*^ | 54 (26.6) | 35,011 (7.59) | <0.001 |
| HFNC oxygen or NIV | 20 (9.85) | 7,383 (1.60) | <0.001 |
| Mechanical ventilation | 6 (2.96) | 3,866 (0.84) | 0.008 |
| ^*^Conventional oxygen therapy refers to oxygen supplementation that does not include HFNC, NIV, or mechanical ventilation, such as the use of nasal prongs or simple oxygen masks.  Abbreviations: PTB, pulmonary tuberculosis; HFNC, high flow nasal cannula; NIV, noninvasive ventilation. | | | |

| **Supplementary table 3.** Logistic regression analysis of the impact of pulmonary tuberculosis on outcomes in COVID-19 patients before propensity score matching. | | |
| --- | --- | --- |
|  | OR (95% CI) | p value |
| **Mortality** |  |  |
| COVID-19 in hospital | 3.01 (1.64-5.51) | <0.001 |
| 30 days | 2.67 (1.66-4.31) | <0.001 |
| 90 days | 2.56 (1.66-3.97) | <0.001 |
| **Severity** |  |  |
| No oxygen | 0.44 (0.32-0.60) | <0.001 |
| Oxygen supply | 2.29 (1.66-3.15) | <0.001 |
| Conventional oxygen therapy^*^ | 1.83 (1.30-2.56) | <0.001 |
| HFNC oxygen or NIV | 2.20 (1.36-3.58) | 0.001 |
| Mechanical ventilation | 1.06 (0.46-2.42) | 0.894 |
| ^*^Conventional oxygen therapy refers to oxygen supplementation that does not include HFNC, NIV, or mechanical ventilation, such as the use of nasal prongs or simple oxygen masks.  Abbreviations: OR, odds ratio; CI, confidence intervals, HFNC, high flow nasal cannula; NIV, noninvasive ventilation. | | |

| **Supplementary table 4.** Multivariable logistic regression analysis of the impact of vaccine administration on outcomes in patients with pulmonary tuberculosis and COVID-19 co-infection. (N=203). | | |
| --- | --- | --- |
|  | aOR (95% CI)^*^ | p value |
| **Mortality** |  |  |
| COVID-19 in hospital | 0.32 (0.01-1.31) | 0.113 |
| 30 days | 0.60 (0.20-1.64) | 0.318 |
| 90 days | 0.46 (0.18-1.19) | 0.110 |
| **Severity** |  |  |
| No oxygen | 3.01 (1.51-5.97) | 0.002 |
| Oxygen supply | 0.33 (0.17-0.66) | 0.002 |
| Conventional oxygen therapy**^†^** | 0.54 (0.27-1.10) | 0.088 |
| HFNC oxygen or NIV | 0.24 (0.08-0.75) | 0.014 |
| Mechanical ventilation | 1.00 (0.18-5.50) | 0.996 |
| **^*^**Adjusted for age, sex, income quartile, and Charlson comorbidity index score.  **^†^**Conventional oxygen therapy refers to oxygen supplementation that does not include HFNC, NIV, or mechanical ventilation, such as the use of nasal prongs or simple oxygen masks.  Abbreviations: aOR, adjusted odds ratio; CI, confidence intervals, HFNC, high flow nasal cannula; NIV, noninvasive ventilation. | | |

| **Supplementary table 5.** The proportion of mortality and disease severity in the study population in which the period of COVID-19 infection was added as a matching variable.^*^ | | | |
| --- | --- | --- | --- |
|  | COVID-19 patients | | p value |
|  | PTB cases  (N =203) | Matched non-PTB controls  (N =812) |  |
| **Mortality,** n (%) |  |  |  |
| COVID-19 in hospital | 13 (6.40) | 13 (1.6) | <0.001 |
| 30 days | 23 (11.33) | 41 (5.05) | 0.002 |
| 90 days | 31 (15.27) | 68 (8.37) | 0.005 |
| **Severity,** n (%) |  |  |  |
| No oxygen | 123 (60.59) | 577 (71.06) | 0.005 |
| Oxygen supply | 80 (39.41) | 235 (28.94) | 0.005 |
| Conventional oxygen therapy**^†^** | 54 (26.6) | 165 (20.32) | 0.057 |
| HFNC oxygen or NIV | 20 (9.85) | 41 (5.05) | 0.013 |
| Mechanical ventilation | 6 (2.96) | 29 (3.57) | 0.831 |
| ^*^Matching variables included: age, sex, income quartile, COVID-19 vaccine status, Charlson comorbidity index score, and period of COVID-19 infection (month).  **^†^**Conventional oxygen therapy refers to oxygen supplementation that does not include HFNC, NIV, or mechanical ventilation, such as the use of nasal prongs or simple oxygen masks.  Abbreviations: PTB, pulmonary tuberculosis; HFNC, high flow nasal cannula; NIV, noninvasive ventilation. | | | |

| **Supplementary table 6.** Logistic regression analysis of the impact of pulmonary tuberculosis on outcomes in COVID-19 patients in which the period of COVID-19 infection was added as a matching variable.^*^ | | |
| --- | --- | --- |
|  | OR (95% CI) | p value |
| **Mortality** |  |  |
| COVID-19 in hospital | 4.21 (1.92-9.22) | <0.001 |
| 30 days | 2.40 (1.41-4.11) | 0.001 |
| 90 days | 1.97 (1.25-3.11) | 0.004 |
| **Severity** |  |  |
| No oxygen | 0.63 (0.46-0.86) | 0.004 |
| Oxygen supply | 1.60 (1.16-2.20) | 0.004 |
| Conventional oxygen therapy**^†^** | 1.42 (0.996-2.03) | 0.052 |
| HFNC oxygen or NIV | 2.06 (1.18-3.59) | 0.011 |
| Mechanical ventilation | 0.82 (0.34-2.01) | 0.668 |
| ^*^Matching variables included: age, sex, income quartile, COVID-19 vaccine status, Charlson comorbidity index score, and period of COVID-19 infection (month).  **^†^**Conventional oxygen therapy refers to oxygen supplementation that does not include HFNC, NIV, or mechanical ventilation, such as the use of nasal prongs or simple oxygen masks.  Abbreviations: OR, adjusted odds ratio; CI, confidence intervals, HFNC, high flow nasal cannula; NIV, noninvasive ventilation. | | |
